# The Impact of the COVID 19 Pandemic on Psychiatric Hospitalizations in a Portuguese Department: A Retrospective Observacional Study

**DOI:** 10.1192/j.eurpsy.2024.1063

**Published:** 2024-08-27

**Authors:** J. R. Freitas, C. P. Desport, D. O. Martins, M. Santos, C. Fonseca

**Affiliations:** Psiquiatria, Centro Hospitalar e Universitário de Santo António, Porto, Portugal

## Abstract

**Introduction:**

The World Health Organization declared the coronavirus outbreak a pandemic on March 11th 2020. Since then, the containment measures were leading to increasing mental health problems in the general population and worsening of some pre‑existing psychiatric conditions. To our knowledge, there are few studies characterizing the impact of the COVID‑19 pandemic on psychiatric hospitalizations across the world.

**Objectives:**

We aimed to compare the number and characteristics of the hospitalizations in the mental health department of a Portuguese psychiatric hospital from March 2nd 2019 to October 31st 2019 with those that occurred in the same period in 2020.

**Methods:**

We conducted a retrospective observational study including all patients admitted to hospital during these periods (n=805). Sociodemographic data, clinical characteristics and information about the context of hospitalization were collected. Statistical analysis was performed using t Student Test, Mann‑Whitney and Chi‑square.

**Results:**

In the pandemic period there was a marked reduction in the number of psychiatric hospitalizations. There was a statistically significant difference in the median length of stay and in the percentage of involuntary hospitalizations between the two periods. In 2019, the most frequent International Classification of Diseases (10th Revision) diagnostic categories were F30‑F39 (mood disorders) and in 2020 were F20‑F29 (schizophrenia, schizotypal and delusional disorders).

**Conclusions:**

The reorganization of services and the decrease in admissions through the emergency department may explain these results.

**Disclosure of Interest:**

None Declared

